# Toxoplasmosis-associated hemophagocytic lymphohistiocytosis during tumor-infiltrating lymphocyte therapy

**DOI:** 10.1128/asmcr.00070-26

**Published:** 2026-08-06

**Authors:** Tariq Odeh, Priscilla Quach, Amin Mostofizadeh, Julia D. Hankins, Robert Hamilton-Seth, Chelsea Gorsline

**Affiliations:** 1 Department of Internal Medicine, Division of Pulmonary, Critical Care, and Sleep Medicine, University of Kansas Medical Center 21638, Kansas City, Kansas, USA; 2 Department of Pathology, University of Kansas Medical Center 21638, Kansas City, Kansas, USA; 3 Department of Internal Medicine, Division of Infectious Diseases, University of Kansas Medical Center 21638, Kansas City, Kansas, USA; Pattern Bioscience, Austin, Texas, USA

**Keywords:** HLH, TIL therapy, immunocompromised, toxoplasmosis, opportunistic infection

## Abstract

**Background:**

Hemophagocytic lymphohistiocytosis (HLH) is a severe hyperinflammatory syndrome triggered by infections, malignancies, or autoimmune disorders. Tumor-infiltrating lymphocyte (TIL) therapy requires lymphodepleting chemotherapy and interleukin-2, producing profound immunosuppression. While HLH-like syndromes have been characterized after chimeric antigen receptor T-cell therapy, HLH has not been reported in the setting of TIL therapy. *Toxoplasma gondii* is a recognized but uncommon parasitic trigger of secondary HLH.

**Case Summary:**

A 68-year-old male with metastatic melanoma undergoing TIL therapy presented with altered mental status. He had chronic lymphopenia and reported gastrointestinal intolerance to trimethoprim-sulfamethoxazole, documented as a sulfonamide allergy without formal testing, limiting prophylaxis to inhaled pentamidine, which has no anti-*Toxoplasma* activity. On admission, he was febrile (39.3°C) with pancytopenia, hypofibrinogenemia (44 mg/dL [normal 200–400]), hyperferritinemia (>30,000 ng/mL [normal 30–300]), hypertriglyceridemia (410 mg/dL [normal 150]), and elevated soluble CD25 (6,979.4 pg/mL [normal 175.3–858.2]); HScore was 283 (>99% probability of HLH). Bone marrow biopsy revealed bradyzoites, tachyzoites, pseudocysts, and hemophagocytosis. Disseminated toxoplasmosis was confirmed by positive serum *Toxoplasma* IgG, negative IgM, and cerebrospinal fluid *Toxoplasma* PCR of 13,700 copies/mL (reference: not detected), with brain MRI demonstrating targetoid enhancing lesions. Treatment with pyrimethamine, sulfadiazine, and leucovorin was initiated, but the patient continued to deteriorate and transitioned to comfort measures.

**Conclusion:**

This represents the first toxoplasmosis-associated HLH during TIL therapy. It underscores the importance of distinguishing drug intolerance from true allergy, ensuring *Toxoplasma*-active prophylaxis in profoundly immunosuppressed patients, and recognizing that HIV-derived CD4 thresholds may not apply to non-HIV immunocompromised populations.

## INTRODUCTION

Hemophagocytic lymphohistiocytosis (HLH) is a severe, rapidly progressing inflammatory syndrome that can be classified as primary or secondary. Secondary HLH often arises in the context of infections, malignancies, or autoimmune disorders (1). Tumor-infiltrating lymphocyte (TIL) therapy, used in cancer treatment, involves the extraction, *ex vivo* expansion, and reinfusion of the patient’s tumor-resident T cells, alongside lymphodepleting chemotherapy and interleukin-2 (IL-2) administration to enhance the antitumor immune response (2). While TIL therapy is known to induce lymphopenia, it has not previously been associated with HLH (3). *Toxoplasma gondii* is an obligate intracellular parasite that infects approximately 11% of the U.S. population based on NHANES 2009–2014 seroprevalence data, with higher rates historically and in certain geographic regions (4, 5). Cats are the definitive host, shedding oocysts in feces that can persist in soil for months; humans acquire infection through ingestion of oocysts from contaminated soil or water, or through consumption of undercooked meat containing tissue cysts (6, 7). In immunocompetent hosts, primary infection is typically controlled by cell-mediated immunity, with bradyzoites persisting within tissue cysts in a latent state. In immunocompromised patients, impaired T-cell surveillance allows bradyzoites to reconvert to tachyzoites, the rapidly replicating, tissue-destructive form (2–4 µm × 4–8 µm, crescent-shaped), causing disseminated disease (6, 8). This report discusses a unique case of HLH associated with disseminated toxoplasmosis in a patient with chronic lymphopenia due to TIL therapy, highlighting the intersection of immune dysregulation and opportunistic infections in an immunocompromised host. This case also addresses the complications arising from reported intolerance to trimethoprim-sulfamethoxazole (TMP-SMX), which precluded standard prophylaxis against toxoplasmosis, thus contributing to a gap in current clinical management strategies.

## CASE PRESENTATION

### Patient information

A 68-year-old male with metastatic melanoma, diagnosed 2 years prior, presented after being transferred from an outside facility for altered mental status and visual hallucinations. He lived in a rural “barndominium,” a converted barn with a dirt floor and proximity to animals, including barn cats. This living environment represented classic risk factors for *T. gondii* oocyst exposure, as cats are the definitive host and oocysts shed in feces can persist in soil for prolonged periods (6, 7). The family reported no recent international travel, though their limited contact with the patient left the full travel history uncertain.

The patient’s melanoma was treated with adjuvant pembrolizumab for 9 months approximately 20 months before TIL therapy; however, the disease progressed, and he subsequently received four cycles of nivolumab and relatlimab. Despite this regimen, his melanoma advanced, and TIL therapy was scheduled for Day 0. Preceding TIL therapy, the patient had persistent lymphopenia for 5 months before TIL therapy, which led to initiating prophylactic TMP-SMX, prescribed at 160/800 mg orally three times weekly, 2 months before TIL therapy. However, the patient reported gastrointestinal symptoms after taking only five tablets, which was documented in the medical record as a sulfonamide allergy, leading to its discontinuation. Testing for true IgE-mediated allergy was not performed.

The patient received lymphodepletion with fludarabine and cyclophosphamide on Day −4 (relative to TIL infusion, Day 0), followed by IL-2 from Days +1 to +3. Despite ongoing lymphopenia (absolute lymphocyte count [ALC] near zero), additional prophylactic measures were not taken until inhaled pentamidine (300 mg) administration on the day of TIL therapy. Outpatient oncology follow-ups noted continued lymphopenia; G6PD testing was considered before initiating dapsone 4 weeks after TIL therapy, but dapsone was ultimately not initiated after a CD4 count 6 weeks after TIL therapy, which was found to be 231 cells/µL (normal 491–1,734 cells/µL) with a CD4 percentage of 26.7% (normal 31.5%–62.4%). The decision to defer dapsone appeared to be based on applying HIV-derived CD4 thresholds (200 cells/µL) to this non-HIV patient. Shortly before his final admission on Day +95 (3 months after TIL therapy), his ALC was 0.10 k/µL (normal 1.0–4.8 k/µL).

### Clinical findings

On Day +95, the patient was transferred to our institution for higher-level care due to altered mental status; he was oriented only to his name and the year. His spouse reported normal behavior until the previous day, when he became fatigued and experienced visual hallucinations. On admission, he was febrile (39.3°C) and tachycardic, and labs were significant for pancytopenia with hemoglobin 9.8 g/dL (normal 13.5–16.5 g/dL), white blood cell (WBC) count 1.3 k/µL (normal 4.5–11 k/µL), ALC 0.19 k/µL (normal 1.0–4.8 k/µL), and platelet count 15 k/µL (150–400 k/µL), hypofibrinogenemia with fibrinogen 44 mg/dL (normal 200–400 mg/dL), severe hyperferritinemia with ferritin >30,000 ng/mL (30–300 ng/mL), hypertriglyceridemia with triglycerides 410 mg/dL (normal 150 mg/dL), elevated AST 368 U/L (normal 7–40 U/L), and elevated INR 1.9 (normal 0.9–1.2). Soluble CD25 (sIL-2R) was markedly elevated at 6,979.4 pg/mL (normal 175.3–858.2 pg/mL). Peripheral blood smear showed normocytic anemia with mild anisocytosis, absolute neutropenia with a mild left shift, absolute lymphopenia, and marked thrombocytopenia with normal morphology; the smear was negative for schistocytes, dysplasia, and circulating blasts. Initial imaging included a head CT showing no acute changes and a chest X-ray that was negative for acute cardiopulmonary conditions. No splenomegaly was identified on imaging.

### Diagnostic assessment

A comprehensive infectious workup was performed and included standard aerobic and anaerobic bacterial blood cultures, urinalysis (which met institutional reflex criteria: WBC > 10, positive nitrite, and/or ≥+1 leukocytes), respiratory viral panel, methicillin-resistant *Staphylococcus aureus* (MRSA) nasal PCR, serum Fungitell, cerebrospinal fluid (CSF) *Ehrlichia* PCR, CSF cryptococcal antigen, serum galactomannan, and procalcitonin, all of which were negative except for a mild elevation in procalcitonin at 0.42 ng/mL (>0.25 ng/mL indicates increased likelihood for bacterial infection). Additional tests included serum cytomegalovirus (CMV) PCR, HIV-1 antigen, and HIV-1/2 specific antibodies (fourth-generation antigen/antibody combination assay); acute hepatitis panel (hepatitis A IgM, anti-HBc IgM, HBsAg, and anti-HCV), obtained as part of the standard workup; and Epstein-Barr virus (EBV) PCR, with the only positive finding being a low-level EBV PCR at 77 copies/mL.

Non-infectious evaluations included an electroencephalogram (EEG) and a lumbar puncture (LP). The EEG demonstrated continuous and reactive theta-delta frequency activity with frequent generalized rhythmic delta activity with triphasic morphology, indicative of moderate encephalopathy of non-specific etiology, without epileptiform discharges. The LP revealed CSF glucose 111 mg/dL, total protein 76 mg/dL, WBC 1/µL (reference 5/µL; lymphocytes 60%, monocytes 40%), and Gram stain with no neutrophils and no organisms. The CSF profile was not consistent with bacterial or viral meningitis, and the CSF fungal culture was without growth.

**Fig 1:**
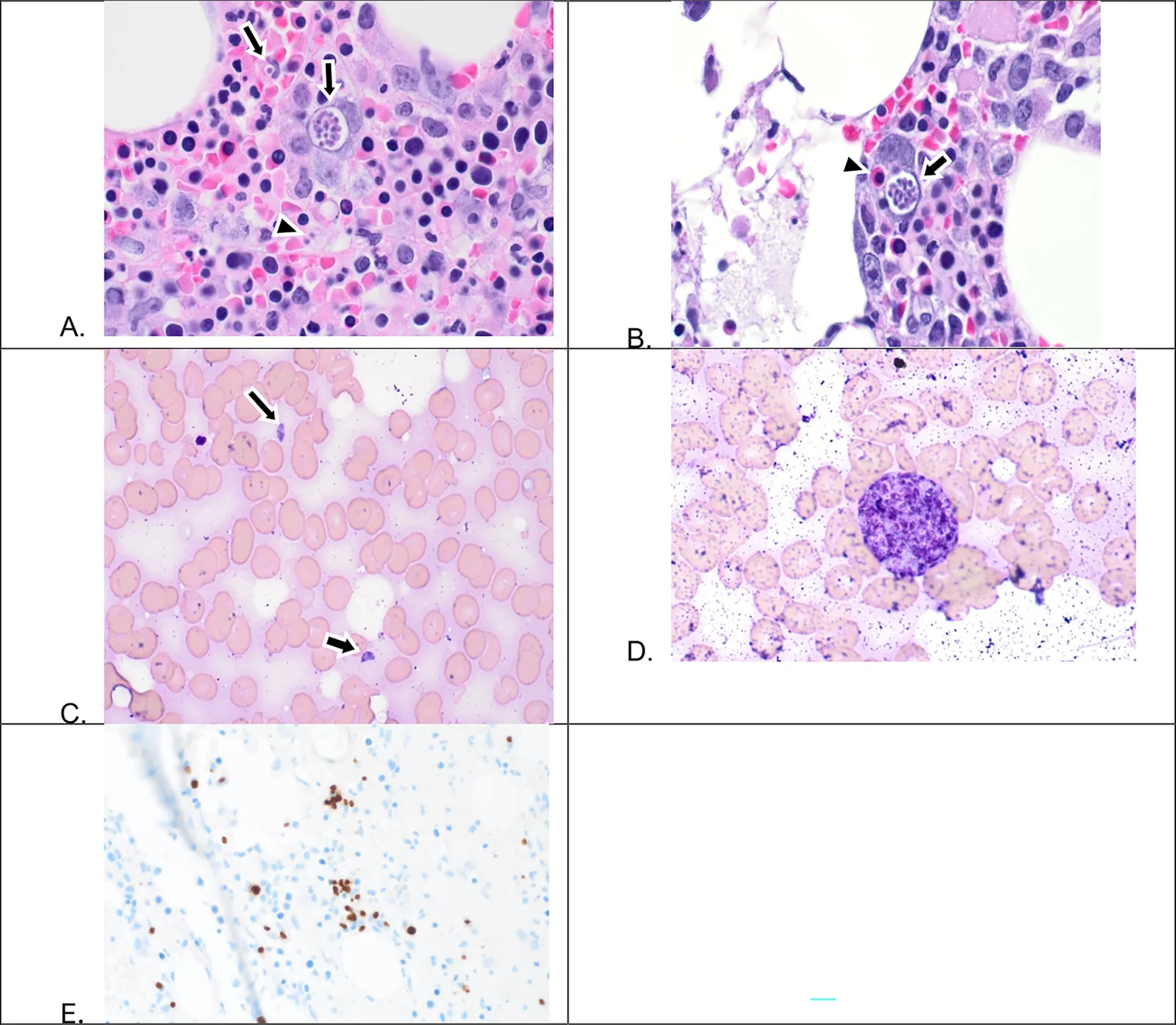
Histopathology of bone marrow. (**A**) Bone marrow core biopsy with intracellular microorganisms (black arrows), extracellular microorganisms (black arrowhead) (Wright-Giemsa stain, 100× oil objective). (**B**) Bone marrow core biopsy with intracellular microorganisms (black arrow) and hemophagocytosis (black arrowhead) occurring in the same cell (Wright-Giemsa stain, 100× oil objective). (**C**) Bone marrow touch preparation with tachyzoites (Wright-Giemsa stain, 100× oil objective). (**D**) Bone marrow touch preparation with pseudocyst. (**E**) Toxoplasma immunostain (50× oil lens).

Hematology was consulted for the patient’s pancytopenia, and a bone marrow biopsy was performed to rule out a potential hematologic disorder, specifically acute promyelocytic leukemia and HLH, or marrow involvement by metastatic melanoma. The biopsy was a dry tap. The preliminary bone marrow report described microorganism infection involving a hypercellular bone marrow (60%–80% cellularity) with trilineage hematopoiesis, mildly increased erythropoiesis, decreased megakaryopoiesis, and 2% blasts. Bone marrow fungal and acid-fast bacilli cultures were without growth. On histologic examination, circular, intracellular forms were seen initially in the bone marrow biopsy, raising the possibility of *Trypanosoma* or *Leishmania* amastigotes, versus the consideration of *Toxoplasma* bradyzoites. The subsequent visualization of plump, oblong forms resembling tachyzoites elsewhere in the section, as well as in accompanying Wright-stained touch preparations (Fig. 1A through D), led our Pathology department to favor *Toxoplasma*, which was confirmed by PCR, immunohistochemistry (Fig. 1E), and serology. The other two differential elements (*Leishmania* and *Trypanosoma*) were not initially favored for several reasons, the first being an incompatible (if limited) travel history. Based on their role in the parasite’s lifecycle, *Leishmania* promastigotes would be an unusual finding in an established infection in the bone marrow. Morphologically, the oblong forms did not appear to be trypanosomes; they lacked visible kinetoplasts and flagella and demonstrated a round-ended shape much more suggestive of tachyzoites.

**Fig 2:**
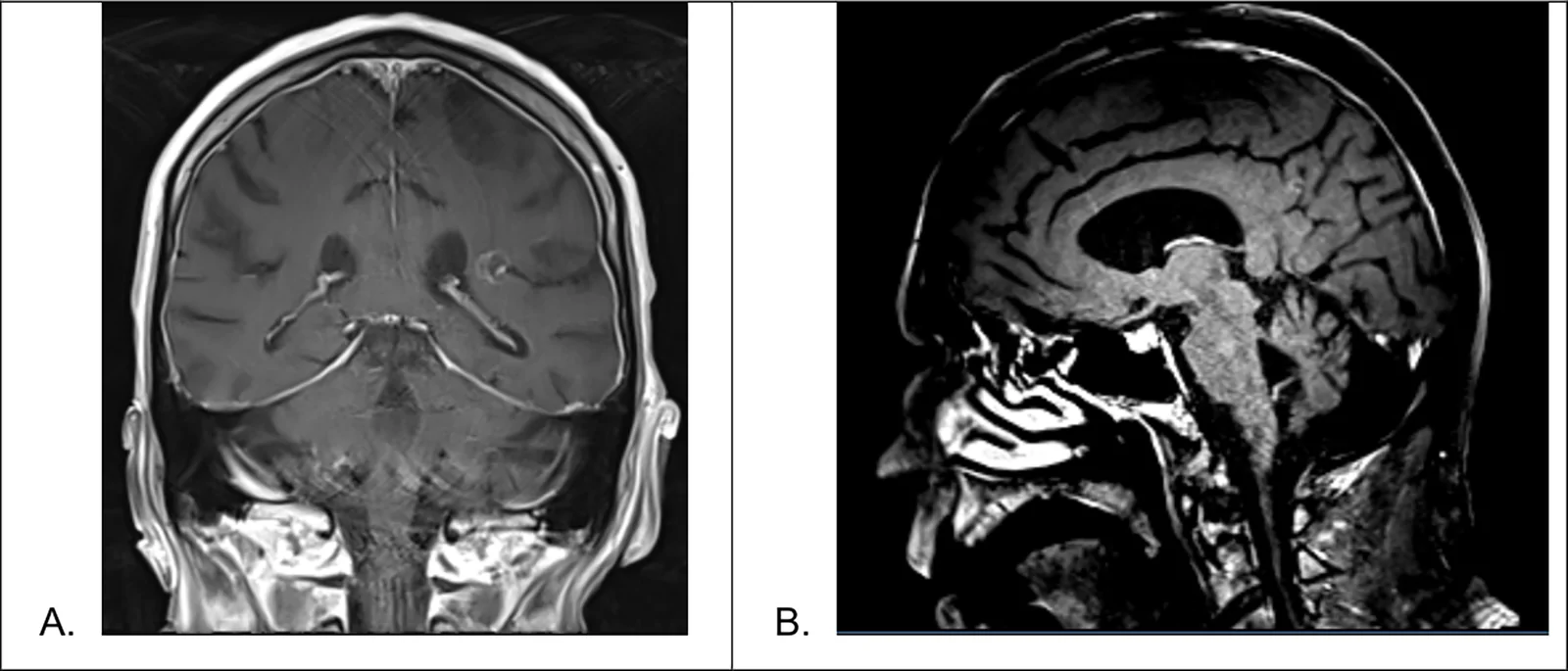
MRI of head. Two lesions were visible on the MRI. (**A**) Small rim-enhancing left posterior insular-perisylvian lesion with eccentric targetoid morphology. (**B**) Tiny rim-enhancing precuneus left parietal lesion.

The infectious disease team was consulted, and brain magnetic resonance imaging (MRI) identified small targetoid enhancing lesions in the left posterior insular and left parietal regions (Fig. 2). Serum *Toxoplasma* IgG by indirect chemiluminescent immunoassay returned positive, and serum *Toxoplasma* IgM was negative. CSF *Toxoplasma gondii* PCR returned at 13,700 copies/mL (reference: not detected). The diagnosis of disseminated toxoplasmosis was therefore based on the convergence of bone marrow morphology, positive serum IgG, and elevated CSF PCR; the *Toxoplasma* immunostain provided definitive confirmation posthumously. Additionally, serum *Trypanosoma cruzi* IgG was negative (0.2 IV; reference ≤ 1.0 IV), and *Leishmania* antibody was negative, further supporting the diagnosis of toxoplasmosis over other morphologic differentials.

In this patient, the HScore was 283 (>99% probability of HLH), based on immunosuppression, fever (39.3°C), absence of organomegaly, 3-lineage cytopenias, ferritin > 6,000 ng/mL, triglycerides > 354 mg/dL, fibrinogen ≤250 mg/dL, AST ≥ 30 U/L, and hemophagocytosis on bone marrow (9). The initial concern for acute promyelocytic leukemia was prompted by the combination of pancytopenia with severe hypofibrinogenemia and elevated INR, which can mimic disseminated intravascular coagulation; however, the peripheral blood smear was negative for circulating blasts and dysplasia, and bone marrow examination showed only 2% blasts, effectively excluding this diagnosis.

### Therapeutic intervention

Treatment with pyrimethamine (200 mg loading dose via nasogastric [NG] tube, then 75 mg/day via NG tube), sulfadiazine (1,500 mg via NG tube every 6 h), and leucovorin (25 mg/day via NG tube) was initiated, prioritizing the critical need for sulfadiazine despite the reported sulfonamide intolerance due to the patient’s critical condition.

### Follow-up and outcomes

The patient’s condition continued to deteriorate despite aggressive care for toxoplasmosis and HLH. He remained intubated. The patient then developed shock, and renal function worsened; however, kidney replacement therapy (i.e., continuous renal replacement therapy) was not pursued given his guarded prognosis. Despite aggressive management, the patient did not show significant improvement. The family, including his spouse, engaged in discussions and reached a consensus that the patient would not wish to continue aggressive care, instead prioritizing comfort and avoiding suffering. The patient eventually transitioned to comfort measures and passed shortly thereafter.

### Timeline of events

See Fig. 3.

**Fig 3:**
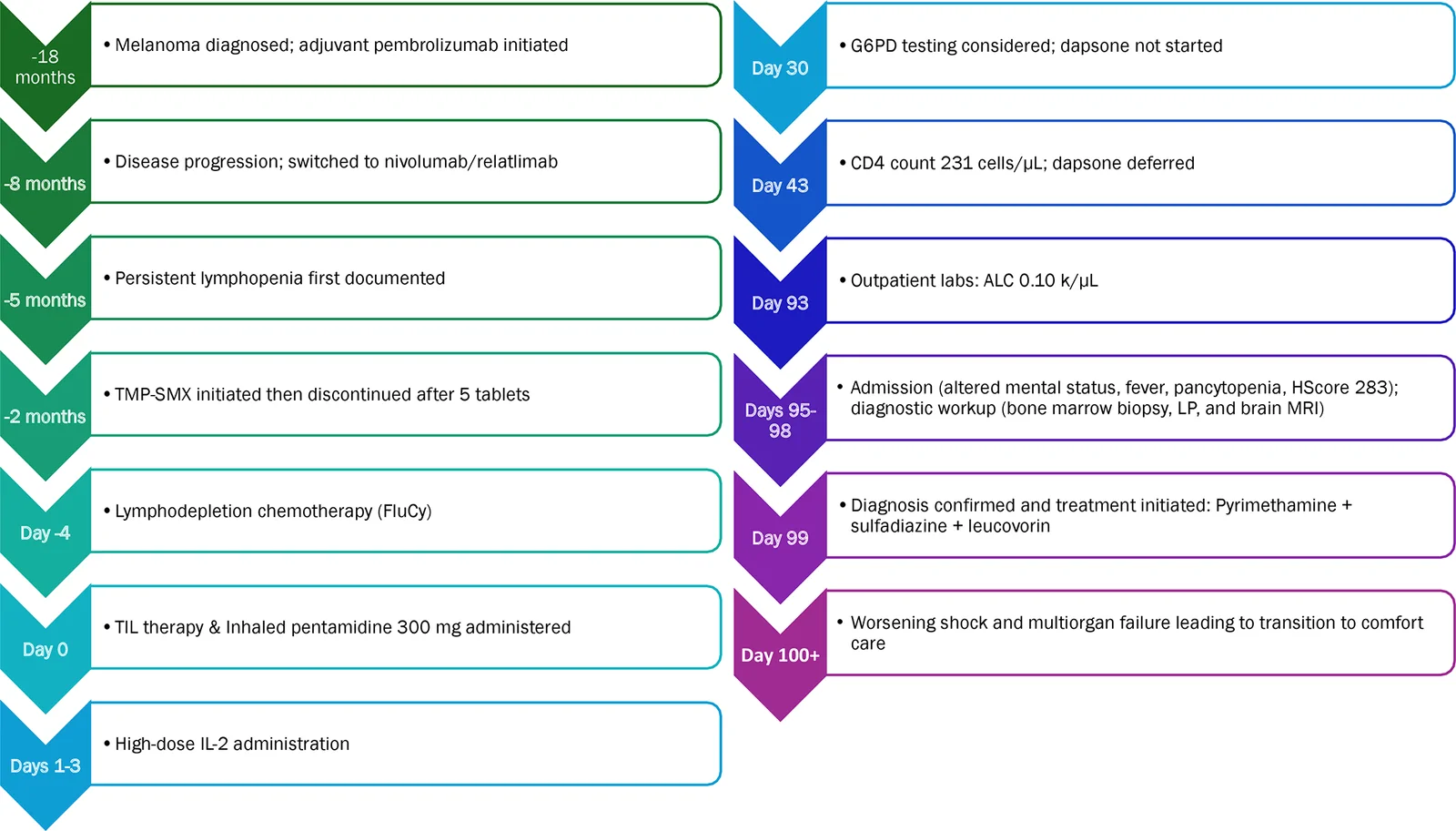
Timeline of clinical events. Day 0 = TIL therapy infusion. Negative values indicate events before TIL infusion; positive values indicate events after TIL infusion. TIL, tumor-infiltrating lymphocyte; TMP-SMX, trimethoprim-sulfamethoxazole; FluCy, fludarabine and cyclophosphamide; IL-2, interleukin-2; G6PD, glucose-6-phosphate dehydrogenase; ALC, absolute lymphocyte count; LP, lumbar puncture; MRI, magnetic resonance imaging.

## DISCUSSION

A review by Henter (1) conceptualizes HLH as a threshold phenomenon in which endogenous factors (residual cytotoxic function and underlying inflammatory disorder) and exogenous factors (immunosuppression and infectious triggers) accumulate until a critical threshold is crossed. This framework is directly applicable to TIL therapy patients: lymphodepleting chemotherapy creates profound immunosuppression, while high-dose IL-2 drives a cytokine-rich milieu. In this patient, the convergence of treatment-induced immunosuppression and *Toxoplasma* reactivation was the most likely trigger for crossing the HLH threshold. Multiple factors contributed to the cumulative immunosuppression: nivolumab/relatlimab-associated lymphopenia (10), fludarabine/cyclophosphamide lymphodepletion (11), and TIL therapy itself.

The nivolumab/relatlimab prescribing information lists decreased lymphocytes in 32% of treated patients (12), and notably, one of three treatment-related deaths in the RELATIVITY-047 trial was attributed to HLH, highlighting the immunologic risks of this combination (10). Fludarabine combined with cyclophosphamide produces prolonged T-lymphocytopenia (13), with CD4 counts dropping to a median of approximately 198/µL and showing minimal recovery over time, directly paralleling this patient’s course (14).

All patients receiving TIL therapy achieve grade 4 lymphopenia with median ALC nadirs as low as 0.005 k/µL, creating a window of vulnerability (3). While *Toxoplasma* was the most likely trigger, other potential contributors to HLH in this patient include the lymphodepleting chemotherapy (fludarabine and cyclophosphamide), IL-2, and the underlying malignancy.

Parasitic infections, including *T. gondii*, *Leishmania*, and *Plasmodium*, are recognized but uncommon triggers of secondary HLH cases (15). A recent systematic review by Shahini et al. (15) identified 13 case reports involving 14 patients with concurrent HLH and toxoplasmosis, predominantly in immunocompromised males (79%) aged 26–58 years. The co-presentation occurred most in patients with HIV infection, solid organ transplantation, and allogeneic hematopoietic stem cell transplantation (HSCT), with one case following CAR T-cell therapy. Fever was universal, and diagnostic approaches combined serology (positive in 71%), PCR (positive in 79%), and bone marrow examination. The review emphasizes that hemophagocytosis on bone marrow is non-specific and must be interpreted alongside molecular and serological results, with PCR providing the strongest evidence of active infection (15). An earlier literature review by Washino et al. (16) identified eight prior cases, with most patients dying within 3 weeks of hospitalization; notably, patients diagnosed antemortem and treated survived. Additional cases have been reported after haploidentical stem cell transplantation and renal transplantation (17, 18). The case by Obeid et al. (19) similarly demonstrated *Toxoplasma* vs. *Leishmania* as the morphologic differential on bone marrow biopsy, with immunohistochemistry and next-generation sequencing confirming *T. gondii*. The present case represents the 15th reported case of concurrent toxoplasmosis and HLH and the first in the setting of TIL therapy.

The immunopathogenic mechanism linking toxoplasmosis to HLH involves the IL-12/IFN-γ axis. In immunocompetent hosts, IL-12-driven activation of NK cells and CD4+/CD8+ T cells promotes IFN-γ production, macrophage activation, and effective pathogen clearance. In immunocompromised hosts, impaired cytotoxic activity of CD8+ T cells and NK cells leads to ineffective *T. gondii* clearance, sustained IFN-γ and IL-12 signaling, excessive macrophage activation, and the cytokine storm characteristic of HLH (15).

While immune effector cell-associated HLH-like syndromes have been well-characterized after CAR T-cell therapy (20, 21), no analogous data exist for TIL therapy. The National Comprehensive Cancer Network (NCCN) Guidelines for Prevention and Treatment of Cancer-Related Infections explicitly categorize cellular immunotherapies as high-risk for *Pneumocystis jirovecii* (*PJP*) infection, recommending *PJP* prophylaxis for at least 6 months after initiation of cellular immunotherapy, and extended while receiving immunosuppressive therapy or during persistent lymphopenia (22). Importantly, the NCCN notes that TMP-SMX has activity against *Toxoplasma*, *Nocardia*, and *Listeria* in addition to *PJP*.^22^ A meta-analysis of 670 TIL-treated patients found the pooled probability of severe infections was 8% (23). Fatal infectious complications have been documented, including sepsis-induced multiorgan failure during chemotherapy-induced neutropenia (24).

The patient’s EBV PCR of 77 copies/mL warrants discussion. In EBV-triggered HLH, viral loads are typically in the range of 10⁴–10⁶ copies/mL, and multivariate analysis has identified EBV-DNA > 10,000 copies/mL as an independent risk factor for adverse outcomes in EBV-triggered HLH (25, 26). A classification system by Kelesidis et al. (27) assigns only 1 point for EBV loads 5,000 copies/mL and subtracts points when alternative etiologies for HLH exist. The low-level EBV viremia in this patient most likely represents incidental reactivation in the setting of profound immunosuppression rather than a causative trigger for HLH, further supporting *Toxoplasma* as the primary trigger.

In this patient, the serologic pattern of positive *Toxoplasma* IgG with negative IgM was consistent with reactivation of latent infection rather than acute primary infection, as IgM is typically produced during primary infection and disappears within approximately 6 months (6). However, serology in immunocompromised patients may be unreliable due to impaired antibody production, and a negative IgM does not exclude primary infection in this setting (28).

The patient’s CD4 count of 231 cells/µL, measured 6 weeks before presentation, was above the traditional 200 cells/µL threshold used in HIV guidelines for initiating toxoplasmosis prophylaxis. However, this threshold is derived from HIV populations and may not directly apply to non-HIV immunocompromised patients. In allo-HSCT recipients, 27 of 29 patients with toxoplasmosis had CD4 < 200 cells/µL, but cases above this threshold do occur (29). Furthermore, the patient’s ALC was 0.10 k/µL shortly before admission, suggesting further immunologic decline after the CD4 measurement. The ECIL-9 guidelines recommend extending prophylaxis during “severe CD4 lymphopenia” without specifying a strict cutoff for non-HIV patients and recommend prophylaxis based on the immunosuppressive regimen and serologic status rather than CD4 count alone (28). PCR (blood, CSF, and BAL) is the preferred diagnostic modality for toxoplasmosis in immunocompromised patients, with sensitivity of approximately 89% in post-transplant toxoplasmosis. Serology may be unreliable due to impaired antibody production. Tissue biopsy with immunohistochemistry remains the gold standard for proven disease (28, 30).

The diagnosis of HLH in TIL-treated patients is particularly challenging because the expected toxicity profile, pancytopenia, elevated ferritin, hepatic dysfunction, and coagulopathy, overlaps substantially with HLH diagnostic criteria. The HLH-2004 criteria were developed for pediatric populations, and the HScore, while validated in adults, has not been specifically validated in patients receiving cellular therapies (20, 31). As detailed in the Diagnostic Assessment, this patient’s HScore of 283 (>99% probability of HLH) supported the diagnosis, though the overlap between expected TIL therapy toxicities and HLH criteria underscores the need for validated diagnostic tools in this population. The NCCN notes that there is “no agreed upon grading scale” for HLH-like syndromes in the immunotherapy context (22). The optimized HLH inflammatory (OHI) index, combining soluble CD25 > 3,900 U/mL and ferritin > 1,000 ng/mL, has been proposed as a simplified diagnostic tool with high specificity (81%) for malignancy-associated HLH and is independently predictive of mortality (HR 4.3) (32). In this patient, both OHI criteria were met (sIL-2R 6,979.4 pg/mL and ferritin > 30,000 ng/mL). This tool may be useful in the TIL therapy setting where baseline cytopenias confound traditional criteria.

Infection-triggered HLH carries a mortality rate of approximately 39%–58% in adult cohorts (33, 34). Critically, HLH induced by intracellular infections such as *Toxoplasma* typically responds to specific antimicrobial treatment, and aggressive HLH-directed therapy based on HLH-94/HLH-2004 protocols (including etoposide) should typically be avoided in favor of prioritization of pathogen-directed therapy (1). Elevated soluble IL-2 receptor, thrombocytopenia, and a history of malignant disease are associated with adverse outcomes in infection-triggered HLH (34). Shahini et al. (15) similarly recommend a low threshold for initiating targeted anti-*Toxoplasma* therapy when clinical suspicion is high, while also evaluating other possible triggers and co-infections.

Lastly, this case highlights the critical importance of distinguishing true drug allergy from drug intolerance. The patient’s gastrointestinal symptoms after five tablets of TMP-SMX were documented as a sulfonamide allergy without formal allergy testing, leading to discontinuation of the most effective prophylactic agent. The 9th European Conference on Infections in Leukemia (ECIL-9) guidelines identify TMP-SMX prophylaxis failure (including due to intolerance) as a major risk factor for toxoplasmosis in immunocompromised patients (28). In this patient, the only prophylaxis administered was inhaled pentamidine, which provides coverage against *PJP* but has no activity against *Toxoplasma* (35). The decision to defer dapsone based on a CD4 count of 231 cells/µL, applying HIV-derived thresholds to a non-HIV patient, left the patient without any *Toxoplasma*-active prophylaxis during a period of profound immunosuppression. The NCCN explicitly recommends considering TMP-SMX desensitization in patients who require *PJP* prophylaxis but report intolerance and lists atovaquone as an alternative with activity against both *PJP* and *Toxoplasma* (22). TMP-SMX is the preferred agent for secondary *PJP* prophylaxis (36), and as many as 70% of previously intolerant patients can tolerate reinstitution through gradual dose escalation (37). For patients who are *Toxoplasma*-seropositive and cannot take TMP-SMX, dapsone + pyrimethamine + leucovorin is recommended as an alternative with dual *PJP/Toxoplasma* coverage, whereas aerosolized pentamidine and dapsone alone have no activity against *Toxoplasma* (35, 36).

This case has several strengths and limitations that merit discussion. It provides the first documentation of toxoplasmosis-associated HLH in the setting of TIL therapy, expanding the known clinical spectrum of both conditions. The diagnosis was supported by multimodal confirmation including bone marrow morphology, serology, CSF PCR, brain MRI, and posthumous immunohistochemistry. The detailed documentation of the prophylaxis gap and the clinical decision-making surrounding TMP-SMX intolerance and CD4 threshold application provides actionable lessons for clinicians managing profoundly immunosuppressed patients.

As a single case report, the findings cannot be generalized to all TIL therapy recipients. The patient’s travel history was incomplete due to limited family contact, introducing uncertainty regarding the source and timing of *Toxoplasma* acquisition. The exact timing of *Toxoplasma* reactivation relative to TIL therapy cannot be determined. Immunohistochemical confirmation was obtained posthumously, and no autopsy was performed to fully characterize the extent of disseminated disease. Additionally, the relative contributions of the multiple immunosuppressive agents (nivolumab/relatlimab, fludarabine/cyclophosphamide, IL-2, and TIL therapy itself) to the development of HLH cannot be individually delineated.

From the family’s perspective, the patient’s wife, who served as his legal guardian during hospitalization, and his son were closely involved throughout his care. They expressed that the rapid deterioration from his baseline was unexpected and distressing, as the patient had been functioning independently until the day before admission. The family appreciated the multidisciplinary approach involving critical care, hematology, pathology, and infectious disease teams in pursuing the diagnosis. They noted that the complexity of his treatment course, spanning multiple immunotherapy regimens and TIL therapy, made it difficult for them to fully understand the degree of immune vulnerability he faced. In reflecting on the clinical course, the family expressed a hope that sharing his case might help other patients in similar situations receive earlier protective measures against infections. The family provided informed consent for publication of this case report with the understanding that it could contribute to improved care for future patients undergoing similar therapies.

This case report describes the first documented instance of toxoplasmosis-associated HLH occurring in a patient receiving TIL therapy for metastatic melanoma, representing the 15th reported case of concurrent toxoplasmosis and HLH in the literature. While NCCN guidelines address prophylaxis for CAR T-cell therapy, comparable granularity for TIL therapy is lacking (22). It highlights a critical gap in current clinical practice. Gastrointestinal intolerance to TMP-SMX should be distinguished from true allergy through formal evaluation, and when TMP-SMX cannot be used, alternative agents with anti-*Toxoplasma* activity (such as atovaquone or dapsone plus pyrimethamine plus leucovorin) should be initiated rather than agents without *Toxoplasma* coverage (such as aerosolized pentamidine alone). The NCCN recommends that for patients at high risk, “a careful reassessment of the allergy is recommended” when allergy history limits prophylactic options (22). Additionally, HIV-derived CD4 thresholds for initiating prophylaxis should not be directly applied to non-HIV immunocompromised patients; prophylaxis decisions should be guided by the immunosuppressive regimen and clinical context.

## Data Availability

No new data were generated or analyzed in support of this research.
